# Regulation of Obesity by Antiangiogenic Herbal Medicines

**DOI:** 10.3390/molecules25194549

**Published:** 2020-10-04

**Authors:** Soon Shik Shin, Michung Yoon

**Affiliations:** 1 Department of Formula Sciences, College of Oriental Medicine, Dongeui University, Busan 47340, Korea; ssshin@deu.ac.kr; 2 Department of Biomedical Engineering, Mokwon University, Daejeon 35349, Korea; yoon60@mokwon.ac.kr

**Keywords:** angiogenesis, adipose tissue growth, obesity, MMP, medicinal herb, *Panax ginseng*, *Melissa officinalis*

## Abstract

Obesity is the result of an energy imbalance caused by an increased ratio of caloric intake to energy expenditure. In conjunction with obesity, related metabolic disorders, such as dyslipidemia, atherosclerosis, and type 2 diabetes, have become global health problems. Obesity progression is thought to be associated with angiogenesis and extracellular matrix (ECM) remodeling. Angiogenesis occurs in growing adult adipose tissues, which are similar to neoplastic tissues. Adipose tissue is highly vascularized, and each adipocyte is nourished by an extensive capillary network. Adipocytes produce proangiogenic factors, such as vascular endothelial growth factor A and fibroblast growth factor 2, which promote neovascularization within the adipose tissue. Furthermore, matrix metalloproteinases (MMPs), including MMP-2 and MMP-9, play important roles in adipose tissue development and microvessel maturation by modifying the ECM. Thus, modulation of angiogenesis and MMP activity provides a promising therapeutic approach for controlling human obesity and its related disorders. Over the past decade, there has been a great increase in the use of alternative treatments, such as herbal remedies, for these diseases. This review will focus on the role of angiogenesis in adipose tissue growth and the regulation of obesity by antiangiogenic herbal medicines.

## 1. Introduction

Obesity is characterized by increased adipose tissue mass that results from both increased adipocyte number (hyperplasia) and size (hypertrophy) [1]. Development of obesity is associated with extensive modifications in adipose tissue, which involves adipogenesis, angiogenesis, and remodeling of the extracellular matrix (ECM) [2].

Angiogenesis is defined as the formation of new blood vessels from preexisting vessels. It is a fundamental requirement for the survival of new tissues in embryonic development as well as for wound healing, placental development, and cyclical changes within the endometrium in the mature adult female [3]. However, angiogenesis is also part of the underlying pathological processes of all major diseases of the developed world. It is a prominent feature of cancer, atherosclerosis, diabetes, rheumatoid arthritis, and proliferative retinopathy [4,5,6]. Interestingly, the formation of new blood vessels is also required for the growth and development of adipose tissue to provide oxygen and nutrients to adipocytes [7,8].

Similar to neoplastic tissues, angiogenesis occurs in growing adult adipose tissues [2]. Adipose tissue can expand and contract throughout life, whereas most tissues do not typically grow throughout adulthood, and the supporting vasculature is quiescent [9]. Adipose tissue is highly vascularized, and each adipocyte is nourished by an extensive capillary network [2,10,11]. Growing adipocytes produce proangiogenic factors, such as vascular endothelial growth factor A (VEGF-A) and fibroblast growth factor 2 (FGF-2), which contribute to the formation of new blood vessels within adipose tissue [7,12,13]. Therefore, it is suggested that the growth and expansion of adipose tissue depends on angiogenesis and may be inhibited by angiogenesis inhibitors. This is supported by reports that treatment with angiogenesis inhibitors results in weight reduction and adipose tissue loss, demonstrating that adipose tissue mass can be regulated by its vasculature [14,15,16]. 

Prominent alterations in ECM remodeling have also been observed during adipose tissue growth. Two types of proteolytic systems, the plasminogen/plasmin (fibrolytic) and matrix metalloproteinase (MMP) systems, have been implicated in tissue remodeling via degradation of the ECM components or via activation of adipocyte growth factors [17,18,19]. The MMP system plays important roles in the development of adipose tissue and microvessel maturation by modulating the ECM [18,20]. Increasing evidence suggests that endogenous and exogenous MMPs regulate adipogenesis [20,21,22]. Indeed, it is well established that ECM degradation represents the first step in the angiogenic process and that MMP-2 and MMP-9 are necessary for this event [23], indicating that angiogenesis and the MMP system have synergistic actions in the regulation of adipose tissue growth.

In addition to the link between angiogenesis and obesity, it has been suggested that obesity-associated inflammation promotes angiogenesis and cancer. The obese state is associated with white adipose tissue dysfunction, including adipocyte hypertrophy, adipocyte death, macrophage infiltration, and elevated inflammatory cytokines [24,25] Chronic inflammation leads to the development and progression of several cancers, such as colorectal, gastric, breast, lung, and liver [26,27,28,29,30]. Elevated levels of inflammatory cytokines are associated with increased risk of cancer [27,31,32]. Consistent with high levels of inflammatory cytokines and macrophages, vascular permeability is enhanced in obese white adipose tissue, which facilitates the extravasation of tumor cells through the vessel wall, contributing to metastasis in obesity [33,34]. The tumor-promoting effects of obesity may occur via adipose inflammation. Moreover, activated macrophages produce potent proangiogenic factors, including tumor necrosis factor α (TNFα) and interleukin 1β (IL-1β) [32,35]. Thus, adipose inflammation during obesity contributes to increased angiogenesis and cancer.

Based on our previously published results that demonstrate the actions of antiangiogenic herbs against obesity, this review will discuss the regulatory role of angiogenesis in adipose tissue and the use of antiangiogenic herbal medicines for the regulation of adipose tissue growth.

## 2. Angiogenesis and Adipose Tissue Growth

Adipose tissue is primarily a site for fat storage, but it also serves as an endocrine gland that secretes hormones, angiogenic factors, growth factors, cytokines, and free-fatty acids. Adipose tissue consists of diverse cell populations, including preadipocytes, mature adipocytes, adipose stromal cells, endothelial cells, pericytes, fibroblasts, and inflammatory cells. 

### 2.1. Adipose Tissue Vasculature

Adipose tissue exhibits extensive vascularity, and each adipocyte is surrounded by an extensive capillary network. The adipose vasculature supplies nutrients and oxygen to growing adipocytes by simultaneously increasing the size and number of new blood vessels. These vessels also support the infiltration of inflammatory cells and remove waste products. In addition to the production of adipokines by adipocytes, activated endothelial cells also produce various growth factors and cytokines, and fenestrated vessels play an essential part in the local and systemic effects of these factors on the adipose tissue [36]. Furthermore, accumulating evidence shows that capillary endothelial cells communicate with adipocytes via paracrine signaling pathways, extracellular components, and direct cell–cell interactions [11,37,38].

The growth and differentiation of adipocytes are spatially and temporally associated with angiogenesis [2]. The growth and development of white adipose tissue requires extensive remodeling of the vascular network, primarily that of primitive capillary networks. The expansion of adipose tissue can be supported by both neovascularization (for adipocyte hyperplasia) as well as dilation and remodeling of existing capillaries (for adipocyte hypertrophy). Hyperplasia of brown adipose critically depends on angiogenesis because it requires rapid activation of mitosis in precursor adipocytes and endothelial cells for capillary development [39]. It is likely that activation of angiogenesis in white adipose tissue promotes obesity [40]. In contrast to white adipose tissue, active angiogenesis in brown adipose tissue would stimulate the energy expenditure, leading to a lean phenotype. 

To adapt to the changes in volume and metabolic rates of adipose deposits, adipose vasculature requires continuous regulation by several angiogenic modulators. Adipocytes seem to regulate angiogenesis by both cell–cell contact and adipokine secretion [7,11]. Conditioned medium from differentiated 3T3 adipocytes and tissue homogenates of omental adipose tissues induce angiogenesis in the chick chorioallantoic membrane and in the mouse cornea [41,42,43]. Both white and brown adipose tissues produce several proangiogenic growth factors, such as VEGF-A and FGF-2, in addition to antiangiogenic factors, such as thrombospondin-1 (TSP-1). These adipose tissues also produce other angiogenic modulators, including leptin and adiponectin, and their expression ratio determines the angiogenic phenotype of the adipose tissue [4,7,8]. During the differentiation of 3T3-F442A preadipocytes into mature adipocytes, proangiogenic factors are upregulated, whereas TSP-1 and TSP-2 are transiently downregulated [13]. In addition to adipocytes, other types of cells contribute to angiogenesis modulation, including preadipocytes, fibroblasts, endothelial cells, resident macrophages, other inflammatory cells, and stromal cells [44]. 

Adipose tissue growth is deeply associated with the remodeling of ECM. As adipose tissues expand during obesity progression, ECM remodeling and reorganization are essentially required to provide enough spaces for adipocytes to be enlarged (hypertrophy) and to adapt to the formation of new adipocytes from precursor cells (hyperplasia) [45]. ECM components in adipose tissue consist mainly of collagens, fibronectin, and laminin [46]. Additionally, several components, such as TSP-1, MMPs, tissue inhibitors of MMPs (TIMPs), a disintegrin and metalloproteinase (ADAMs), ADAM with thrombospondin motifs (ADAMTS), osteopontin, hyaluronan, and elastin, function as the modulators of ECM remodeling and adipose tissue expansion [47,48]. This process also helps to form new blood vessels that are important for healthy adipose tissue expansion and allows hypoxia that induces chronic low-grade inflammation and fibrosis [49]. Indeed, MMP-9 is able to release matrix-bound vascular endothelial growth factor (VEGF), thus indirectly inducing angiogenesis [50].

### 2.2. Proangiogenic Factors

**Figure 1:**
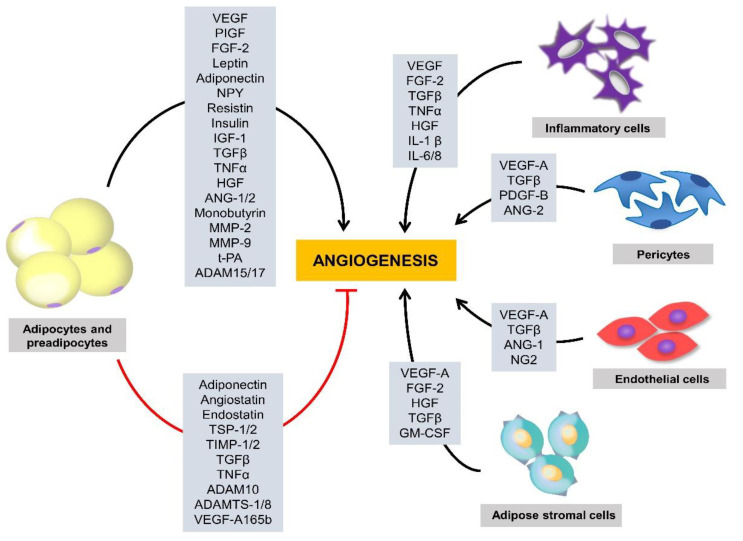
Regulation of adipose tissue angiogenesis by multiple factors. A variety of cells in adipose tissues, including preadipocytes, adipocytes, adipose stromal cells, pericytes, and endothelial cells, contribute to the production of multiple angiogenic stimulators and inhibitors that regulate adipose tissue angiogenesis. ADAM, a disintegrin and metalloproteinase; ADAMTS, ADAM with thrombospondin (TSP) motif; ANG, angiopoietin; FGF, fibroblast growth factor; GM-CSF, granulocyte macrophage colony-stimulating factor; HGF, hepatocyte growth factor; IL, interleukin; IGF-1, insulin-like growth factor 1; MMP, matrix metalloproteinase; NG2, neuron glial antigen 2; NPY, neuropeptide Y; PDGF, platelet-derived growth factor B; PlGF, placental growth factor; TGFβ, transforming growth factor β; TIMP, tissue inhibitor of MMP; TSP, thrombospondin; TNFα, tumor necrosis factor α; t-PA, tissue-type plasminogen activator; VEGF, vascular endothelial growth factor.

Angiogenesis is controlled by an elaborate balance between proangiogenic and antiangiogenic molecules (Table 1). Growing adipocytes produce multiple proangiogenic factors, including VEGF, placental growth factor (PlGF), FGF-2, leptin, neuropeptide Y (NPY), resistin, insulin, insulin-like growth factor 1 (IGF-1), transforming growth factor β (TGFβ), TNFα, hepatocyte growth factor (HGF), angiopoietin (ANG)-1 and ANG-2 (Figure 1). Preadipocytes and adipocytes also produce non-protein, small lipid molecules, such as monobutyrin, that have been shown to stimulate in vivo angiogenesis and in vitro microvascular endothelial cell motility [51]. Adipose stromal cells secrete high levels of various proangiogenic factors, including VEGF, FGF-2, HGF, granulocyte macrophage colony-stimulating factor (GM-CSF), and TGFβ [20]. Inflammatory cell recruitment also significantly contributes to adipose neovascularization. For example, activated macrophages produce potent proangiogenic factors, such as TNFα, VEGF, FGF-2, IL-1β, IL-6, and IL-8 [52]. Additionally, pericytes and endothelial cells play crucial roles in angiogenesis. Pericytes secrete VEGF-A, TGFβ, ANG-1, and neuron glial antigen 2 (NG2), whereas endothelial cells produce VEGF-A, TGFβ, ANG-2, and platelet-derived growth factor B (PDGF-B) [53,54].

It is generally accepted that the VEGF/VEGF receptor (VEGFR) system accounts for most of the angiogenic activity in adipose tissues, which makes it an attractive target to reduce obesity [55,56]. Among all bodily adipose tissues that have been examined, visceral adipose tissue expresses the highest levels of VEGF [57,58]. VEGF is the major angiogenic factor produced in the omentum, and it is most likely involved in the underlying mechanism of omentum-induced angiogenesis [57]. Endothelial cells from visceral adipose tissues exhibit a more marked proangiogenic and proinflammatory state than those from subcutaneous adipose tissues [58]. Additionally, infiltrated inflammatory cells and stromal cells of adipose tissues also significantly contribute to VEGF production.

The VEGF family currently includes VEGF-A, -B, -C, -D, -E, -F, and PlGF, which bind in a distinct pattern to three structurally related receptor tyrosine kinases, denoted VEGFR-1, -2, and -3. VEGF-A is a major proangiogenic factor that stimulates the proliferation and migration of endothelial cells, and it prevents the apoptosis of endothelial cells [59]. Five forms of VEGF-A are produced in mice by alternative splicing (VEGF-A121, VEGF-A145, VEGF-A165, VEGF-A189, and VEGF-A206). Several studies indicate that VEGF-A stimulates both physiological and pathological angiogenesis by signaling via VEGFR-2 in a strict dose-dependent manner. VEGF-B also promotes angiogenesis and is implicated in ECM degradation via the regulation of plasminogen activation. VEGF-C and VEGF-D play a crucial role in the lymphatic system via the promotion of lymphangiogenesis [59]. VEGF-E stimulates the proliferation of endothelial cells both in vivo and in vitro via the activation of VEGFR-2 [60]. VEGF-F possesses weak angiogenic activity but strong vascular permeability [61]. Another member of the VEGF family, PlGF, enhances angiogenesis. Functional inactivation of PlGF in mice leads to impaired adipose tissue development, suggesting that other VEGF members also modulate adipogenesis via the vascular system [62].

FGF-2 is a potent stimulator of differentiation, migration, and proliferation of endothelial cells both in vivo and in vitro [63,64]. It also enhances de novo adipocyte differentiation in mice [63]. During angiogenesis, FGF-2 increases VEGF expression [65] and stimulates the synthesis of proteinases, such as collagenase and urokinase-type plasminogen activator (u-PA), and of integrins to form new capillary cord structures [66,67]. It also stimulates the proliferation of fibroblasts that form granulation tissue in wound healing [68]. Furthermore, FGF-2-induced angiogenesis occurs in the absence of inflammation [69,70], which is a characteristic that distinguishes it from many other angiogenic factors, such as VEGF.

Leptin is a hormone secreted by adipocytes that regulates appetite and energy homeostasis. Interestingly, leptin is also a potent proangiogenic factor that promotes endothelial cell migration. Binding of leptin to its receptor on endothelial cells leads to the activation of the signal transducers and activators of transcription 3 (STAT3) pathway as well as enhancement of its DNA-binding activity [71]. In addition to its direct proangiogenic activity, leptin upregulates VEGF expression via the activation of the Janus kinase/STAT3 signaling pathway [72]. Leptin has a synergistic effect on angiogenesis stimulation by modulating both VEGF and FGF-2 [73]. Leptin also induces MMP-2 and MMP-9 activity, which plays a role in ECM remodeling, and acts as an indirect proangiogenic factor or modulator of other known angiogenic factors [74].

NPY is a small peptide that is important in the promotion of adipogenesis and obesity. NPY also induces in vitro and in vivo angiogenesis via the activation of its Y2 receptor, which is expressed on vascular endothelial cells. Y2 receptor-null mice exhibit inhibition of NPY-induced angiogenesis and delayed wound healing [75]. The adipokine, resistin, is an angiogenic factor that stimulates the proliferation, migration, and tube formation of endothelial cells [76]. Insulin enhances proangiogenic factors, such as VEGF, and increases the survival of pericytes; however, it also reduces the expression of antiangiogenic proteins [77,78]. IGF-1 is a prosurvival factor for many cell types, and it promotes angiogenesis in endothelial cells/adipose-derived stem cells coculture system by enhancing the expression of angiogenesis-related growth factors [79,80].

TGFβ is expressed in endothelial cells and pericytes, and it is increased in the adipose tissues of obese mice [81]. TGFβ can positively and negatively regulate angiogenesis in a concentration-dependent manner in endothelial cells [82,83]. Similar to TGFβ, TNFα also has proangiogenic and antiangiogenic activities that most likely depend on the concentration and duration of exposure as well as the cell type [84,85]. TNFα induces in vivo vessel formation at very low doses and stimulates in vitro capillary endothelial cell chemotaxis; however, it inhibits in vitro endothelial cell proliferation [84,85]. Preadipocytes and adipocytes produce high levels of HGF, which is an important proangiogenic factor for vessel growth and remodeling [86]. ANG-2 is a proangiogenic factor that is elevated in overweight and obese individuals [87]. Mice that overexpress ANG-2 show increased subcutaneous adipose tissue vascularization [88]. Similarly, ANG-2 neutralization in wild-type mice fed a high-fat diet (HFD) show reduced subcutaneous adipose tissue vascularization.

PDGF-B released by endothelial cells promotes the recruitment of pericytes, vascular stabilization, and blood vessel maturation [89,90]. NG2 is a suitable pericyte marker and promotes endothelial cell motility and angiogenesis [53,91].

ECM proteolysis is required for cell migration during blood vessel development and also for adipose tissue expansion. MMPs are key factors involved in ECM degradation, and their main actions in adipose tissues include adipogenesis, angiogenesis, and adipose tissue expansion. Changes in MMP expression patterns and activities, as well as in the balance between MMPs and TIMPs, are crucial for ECM remodeling. Currently, 28 MMPs have been identified and classified according to their substrate specificity [92]. However, the MMP expression patterns in adipose tissue are still controversial, which could possibly be due to differences in experimental models and adipose tissue distribution. Increased MMP-2, but not MMP-9, activity occurs in the adipose tissues of a diet-induced obesity mouse model [93,94]. In contrast, the mRNA expression of MMP-3, MMP-11, MMP-12, MMP-13, and MMP-14 is upregulated, whereas that of MMP-7, MMP-9, MMP-16, and MMP-24 is downregulated in the obese mice [95]. In 3T3-L1 and 3T3-F442A preadipocytes, gelatinase (MMP-2 and MMP-9) inhibitors prevent differentiation into adipocytes in a dose-dependent manner, suggesting that MMP-2 and MMP-9 may be necessary mediators of adipocyte differentiation [96,97].

The fibrinolytic system (plasminogen/plasmin) may also be implicated in the proteolytic activity required for adipose tissue development. Plasminogen is converted into the active enzyme, plasmin, by tissue-type plasminogen activator (t-PA), which allows plasmin to degrade fibrin into soluble fibrin-degradation products. Additionally, t-PA also stimulates angiogenesis and VEGF expression in endothelial cells [98]. tPA-deficient mice on a HFD exhibit higher body weights and adipose tissue masses as well as an increased number of endothelial cells than control mice [99], whereas mice deficient in plasminogen exhibit reduced fat accumulation [100]. Proteins within ADAM and ADAMTS families may also contribute to the regulation of angiogenesis and adipogenesis [8]. For example, endothelial cell tube formation is decreased in ADAM15 small interfering RNA-treated endothelial cells, and an overexpression of ADAM17 in endothelial cells downregulates TSP-1 expression [101,102], suggesting that both ADAM15 and ADAM17 can stimulate angiogenesis.

Other components of ECM have been found to stimulate angiogenesis (Table 2). The two major components of ECM are collagen IV and laminins. The interaction of endothelial cells with full length collagen IV promotes angiogenesis [103,104] and intact laminins stimulate proliferation and migration of endothelial cells [105,106,107]. Fibronectin is a ubiquitously expressed ECM protein and strongly associated with endothelial ECM. Fibronectin increases the number of microvascular cells and promotes endothelial cell survival and migration [108,109,110]. Hyaluronan is a widely distributed ECM macromolecule. The partial degradation fragments of hyaluronan promote proliferation and migration of endothelial cells [111]. Osteopontin is an ECM protein expressed in a variety of tissue types. Osteopontin can induce VEGF release and stimulate proliferation and migration of endothelial cells [112,113]. Elastin is also a key protein of ECM. Elastin and bioactive elastin peptides, termed elastokines, not only enhance angiogenesis, but also upregulate proMMP-2 expression and activity [114,115].

### 2.3. Antiangiogenic Factors

Adipose tissue produces several angiogenesis inhibitors, including adiponectin, angiostatin, endostatin, TSP-1, TSP-2, TIMPs, ADAM, ADAMTS, and VEGF-A165b. In contrast to proangiogenic factors, regulation of adipose vessel growth and remodeling by endogenous angiogenesis inhibitors is relatively poorly understood. 

Adiponectin is an adipose-derived adipokine that is significantly decreased in obese animals and humans. Adiponectin levels in endothelial cells are reported to be inversely correlated with in vitro angiogenesis. Adiponectin inhibits endothelial cell proliferation, migration, and angiogenesis via the reduction of MMP-2, MMP-9, and VEGF, via caspase-mediated endothelial cell apoptosis, or via inhibition of autophagy in rhesus choroid-retinal endothelial cells [116,117,118,119]. However, in vivo and in vitro studies have shown that adiponectin promotes migration and tube formation of endothelial cells, VEGF expression, adipose tissue vascularity, and mouse Matrigel plug implantation [120,121,122], suggesting that the relationship between adiponectin and angiogenesis is still unclear. 

Angiostatin is an internal proteolytic fragment of plasminogen, and most crinkle domains of plasminogen inhibit angiogenesis [123]. Angiostatin induces weight reduction in obese *ob/ob* mice, relative to controls [14]. 

The modulation of angiogenesis by TSP-1 and TSP-2 has been extensively studied [124]. TSP-1 and TSP-2 are potent endogenous inhibitors of angiogenesis. They inhibit angiogenesis through direct effects on endothelial cell migration, proliferation, survival, and apoptosis and by antagonizing VEGF and basic FGF (bFGF) activities. 

MMP activity is modulated through interactions with TIMPs. Of the four TIMPs, most can inhibit the activities of all MMPs [125]. TIMP expression analysis in the adipose tissues of obese mice has shown that TIMP-1 mRNA is upregulated with obesity, whereas TIMP-4 mRNA is downregulated and TIMP-2 and TIMP-3 mRNA are not significantly altered [95]. Interestingly, TIMP-1 deficiency decreases body weight and adipose tissue mass, suggesting that TIMP-1 promotes adipose tissue development [126]. An explanation for these inexplicable findings may be that the expression levels of angiogenesis inhibitors may increase to limit further vascular growth when the adipose tissue growth rate plateaus. Consistent with this hypothesis, TSP-1 expression is downregulated in preadipocytes, but it is upregulated in differentiated adipocytes [127]. TSP-1 loss attenuates weight gain and fat accumulation in HFD-fed mice without any significant effects on adipocytes or adipose tissue development [128,129]. Thus, the regulatory role of TSP-1 in adipose tissue angiogenesis warrants further investigation. 

ADAMTS-1 and ADAMTS-8 can inhibit VEGF-induced angiogenesis and suppress FGF-2-induced vascularization [130]. Both factors mediate a greater antiangiogenic response than that of TSP-1 or endostatin, with ADAMTS-1 showing a greater inhibitory capacity than ADAMTS-8. The antiangiogenic activities of ADAMTS-1 and ADAMTS-8 are mediated through their thrombospondin (TSP) motifs. ADAMTS-1 significantly inhibits VEGFR2 phosphorylation with consequent suppression of endothelial cell proliferation [131]. Furthermore, inhibition of ADAM10 induces vessel formation and density in vivo, indicating that ADAM10 may also have a positive effect on sprouting angiogenesis [81]. 

In contrast to the proangiogenic effects of VEGF-A165a, VEGF-A165b, which is a splice variant of the VEGF-A gene, possesses antiangiogenic activity [132,133]. VEGF-A165b inhibits angiogenesis and neovascularization in several types of experimental models.

ECM components have been shown to inhibit angiogenesis. Endostatin is a C-terminal fragment of type XVIII collagen, and *ob/ob* mice receiving endostatin exhibit weight loss or inhibited weight gain [14,134]. Endostatin also prevents diet-induced obesity by inhibiting angiogenesis and adipogenesis [14,135]. N-terminal fragments of type IV collagen, known as arresten, canstatin, and tumstatin, function as potent inhibitors of angiogenesis [103,136]. Similar to collagen IV, proteolytic peptides of laminins may inhibit angiogenesis [105]. Fibronectin and anastellin (the III1-C fibronectin fragment) also decrease blood vessel densities in mice [137]. Native hyaluronan inhibits angiogenesis by decreasing endothelial cell proliferation and migration and capillary tube formation [138] but accelerates bFGF-induced neovascularization in Matrigel plugs assays [139]. Thus, the role of native hyaluronan in angiogenesis needs to be further examined.

## 3. Modulation of Obesity by Antiangiogenic Agents

Newly formed adipose tissue relies on continued angiogenesis to sustain growth. Thus, substantial evidence suggests that various angiogenesis inhibitors can significantly reduce body weight gain and adipose tissue mass, which further indicates the large role of angiogenesis in adipose tissue growth. Several types of angiogenesis inhibitors, such as TNP-470, CKD-732, galardin, and Bay12-9655, thalidomide and its analogs, and VEGFR2 inhibitors, have been shown to inhibit fat mass expansion in mice (Table 3).

TNP-470 is a synthetic analog of the fungal metabolite, fumagillin, which inhibits in vitro endothelial cell proliferation and in vivo angiogenesis [140]. TNP-470 also significantly reduces body weight in obese animal models, such as A^y^, Cpe^fat^, and *ob/ob* mice, and it suppresses 3T3-L1 preadipocyte proliferation [10,14]. HFD-fed C57BL/6J mice also show less vessel growth and weight gain with TNP-470 treatment [16,141]. CDK-732 is a TNP-470 analog that significantly decreases body weight, fat pads, and adipocyte size in various animal models, such as arcuate nucleus lesion mice, *ob/ob* mice, Sprague Dawley rats, and Otsuka Long-Evans Tokushima fatty rats [142].

Galardin and BAY 12-9566 are MMP inhibitors [143]. Galardin significantly reduces gonadal and subcutaneous adipose tissue masses, but not body weight in HFD-fed wild-type mice, suggesting a role of MMP inhibitors in the adipose tissue development [17]. BAY 12-9566 treatment results in weight loss or reduced weight gain in *ob*/*ob* mice relative to controls [14].

Thalidomide and its analogs suppress cell proliferation and angiogenesis, but the use of this drug has been stopped due to its teratogenic effect in humans [144]. Additionally, thalidomide treatment results in weight loss in *ob/ob* mice [14].

VEGFR2 inhibitors can limit diet-induced adipose tissue expansion and adipocyte differentiation during in vivo adipogenesis [55,145].

## 4. Antiobesity Effects of Antiangiogenic Herbal Medicines

Over the past decade, there has been a notable increase in the use of alternative treatments, especially herbal remedies. Herbal extracts or active components have been shown to inhibit angiogenesis and obesity (Table 4).

Curcumin, a major component of turmeric (*Curcuma longa*), suppresses adipogenesis in 3T3-L1 adipocytes as well as angiogenesis and obesity in HFD-fed obese C57BL/6J mice [146]. Curcumin inhibits adipokine-induced angiogenesis of human umbilical vein endothelial cells and reduces 3T3-L1 differentiation. Curcumin supplementation not only decreases the expression of VEGF and VEGFR in obese mice, but also reduces body weight gain, adipocyte, and adipose tissue vascularization.

Green tea catechin, epigallocatechin gallate (EGCG), inhibits endothelial cell tube formation by inhibiting VEGF signaling [147]. EGCG at physiological concentrations interferes with the formation of VEGF and VEGFR2 complexes, leading to decreased angiogenic signaling. EGCG also reduces body weight, epididymal white adipose tissue mass, and lipogenesis gene expression in HFD-fed mice [148]. 

Korean red ginseng (*Panax ginseng*) prevents obesity by inhibiting angiogenesis in HFD-induced obese C57BL/6J mice and *db/db* mice [94,149,150]. Korean red ginseng extract (GE) decreases blood vessel densities in the visceral adipose tissues of obese mice [94,149]. GE decreases VEGF-A and FGF-2 mRNA levels but increases TSP-1 mRNA levels in adipose tissues. GE decreases MMP-2 and MMP-9 mRNA levels but increases the levels of TIMP-1 and TIMP-2 mRNA. Administration of GE suppresses MMP-2 activity in adipose tissues of HFD-fed obese mice. Ginseng and its active components, ginsenosides (GSs), inhibit adipogenesis in 3T3-L1 preadipocytes by regulating MMP-2 and MMP-9 [151]. Among the GSs, Rb1 most effectively inhibits MMP activity. Moreover, the inhibitory actions of GE and GSs on adipogenesis are attenuated by the MMP activator, phorbol 12-myristate 13-acetate. 

The antiangiogenic herbal composition, Ob-X, which is composed of lemon balm (*Melissa officinalis)*, white mulberry (*Morus alba)*, and injin (*Artemisia capillaris)*, reduces adipose tissue growth and development in nutritionally and genetically obese mice and inhibits adipogenesis in 3T3-L1 preadipocytes [152,153,154]. Ob-X inhibits in vitro tube formation of endothelial cells, VEGF-induced microvessel outgrowth in an ex vivo rat aortic ring assay, and the formation of new blood vessels induced by VEGF and bFGF in a mouse Matrigel plug assay [152,153]. Furthermore, visceral adipose tissue sections from Ob-X-treated mice have much lower blood vessel densities than those of untreated mice. Ob-X exerts a specific regulatory effect on genes involved in angiogenesis and the MMP system in adipose tissues. Consistent with the findings above, body weight gain and adipose tissue mass of treated mice are significantly less than those of untreated mice. Ob-X is capable of suppressing adipogenesis and adipocyte-specific gene expression [154]. Ob-X also suppresses MMP-2 and MMP-9 gelatinolytic activities in 3T3-L1 adipocytes.

Lemon balm extract, ALS-L1023, also regulates adipogenesis and obesity by inhibiting angiogenesis and MMP activities in animal models of obesity [93,155,156]. ALS-L1023 inhibits endothelial cell VEGF- and FGF-induced tube formation [155]. Its inhibitory effect on endothelial cell proliferation is comparable to that of TNP-470. ALS-L0123 decreases the number of von Willebrand factor- (a marker of endothelial cells)-positive cells in HFD-fed obese mice. ALS-L1023 reduces the body weights, visceral adipose tissue mass, and average adipocyte sizes of HFD-fed C57BL/6J mice. ALS-L1023 reduced visceral fat mass in phase II human trial by computed tomography analysis (unpublished data) and phase III human clinical trial is in progress. Hepatic lipid accumulation, inflammatory cells, and collagen levels are lower in treated obese female OVX and male mice than in untreated mice [156,157]. ALS-L1023 also alleviates hyperglycemia and glucose intolerance in obese female mice [158]. 

## 5. Conclusions

**Figure 2:**
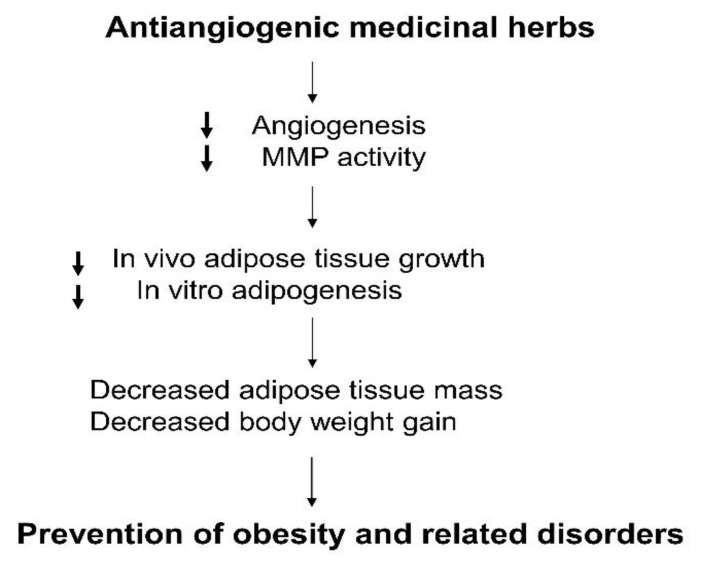
Regulation of obesity by antiangiogenic medicinal herbs.

Obesity is a complex metabolic disorder that is deeply associated with type 2 diabetes, dyslipidemia, atherosclerosis, hepatic steatosis, and cancer. Emerging evidence suggests that modulation of angiogenesis seems to have the potential to reduce fat mass and impair obesity development by regulating adipose tissue vasculature. Interestingly, natural antiangiogenic agents could inhibit obesity and its related disorders. Thus, angiogenesis inhibitors, particularly herbal medicines, may be an attractive pharmacological target for the treatment of obesity and its related metabolic disorders (Figure 2).

## Figures and Tables

**Table 1:** Proangiogenic and antiangiogenic factors and their biological effects on angiogenesis.

Proangiogenic Factor		Antiangiogenic Factor	
Factor	Biological Effect	Factor	Biological Effect
VEGFs	Proliferation and migration of endothelial cells ↑, apoptosis of endothelial cells ↓, Plasminogen activator ↑, ECM degradation ↑, Lymphangiogenesis ↑, Vascular permeability ↑	Adiponectin	Proliferation and migration of endothelial cells ↓, MMP-2, MMP-9, and VEGF expression ↓, apoptosis of endothelial cells ↑
		Angiostatin	Angiogenesis ↓, proliferation of adipocytes ↓
FGF-2	Differentiation, migration, and proliferation of endothelial cells ↑, adipocyte differentiation ↑, angiogenesis ↑, VEGF expression ↑, synthesis of proteinase ↑	TSPs	Migration, proliferation, survival of endothelial cells ↓, apoptosis of endothelial cells ↑, VEGF and bFGF activity ↓
PIGF	Angiogenesis ↑, adipose tissue growth ↑	TIMPs	MMP activity ↓
Leptin	Migration of endothelial cells ↑, VEGF expression ↑, induction of MMP-2 and MMP-9 activity ↑, synergistic effects with VEGF or FGF on stimulation of angiogenesis ↑	MMPs	Angiostatin production ↑
		TGFβ	Proliferation and migration of endothelial cells ↓, tube formation ↓, plasminogen activator ↓, ECM accumulation ↑, apoptosis of endothelial cells ↑
Adiponectin	Migration and tube formation of endothelial cells ↑, VEGF-A expression ↑, adipose tissue vascularity ↑, mouse Matrigel plug implantation ↑	TNFα	Proliferation of endothelial cells ↓
		ADAM10	Vascular sprouting and density ↓
		ADAMTS-1 and -8	VEGF-A-induced angiogenesis ↓, FGF-2-induced vascularization ↓
NPY	Angiogenesis and adipogenesis ↑		
Resistin	Proliferation, migration, and tube formation of endothelial cells ↑	VEGF-A165b	Angiogenesis and neovascularization ↓
Insulin	VEGF expression ↑, antiangiogenic protein expression ↓		
IGF-1	Angiogenesis ↑, MMP expression ↑		
TGFβ	Low dose: VEGF- and bFGF-induced tube formation of endothelial cells ↑,		
TNFα	Low dose: vessel formation ↑, chemotaxis of endothelial cells ↑		
HGF	Vessel growth and remodeling ↑		
ANG-2	Adipose tissue vascularization ↑		
Monobutyrin	Angiogenesis ↑, microvascular endothelial cell mobility ↑		
MMPs	ECM degradation ↑, adipogenesis, angiogenesis, and expansion of adipose tissue ↑, microvessel maturation ↑		
t-PA	VEGF expression ↑, angiogenesis ↑		
ADAM15 and ADAM17	Tube formation of endothelial cells ↑, TSP-1 expression ↓		
PDGF-B	Pericyte recruitment ↑, vascular stabilization ↑, blood vessel maturation ↑		
NG2	Endothelial movement ↑, survival and migration of endothelial cells ↑		

ADAM, a disintegrin and metalloproteinase; ADAMTS, ADAM with TSP motif; ANG, angiopoietin; FGF, fibroblast growth factor; GM-CSF, granulocyte macrophage colony-stimulating factor; HGF, hepatocyte growth factor; IL, interleukin; IGF-1, insulin-like growth factor 1; MMP, matrix metalloproteinase; NG2, neuron glial antigen 2; NPY, neuropeptide Y; PDGF, platelet-derived growth factor B; PlGF, placental growth factor; TGFβ, transforming growth factor β; TIMP, tissue inhibitor of MMP; TSP, thrombospondin; TNFα, tumor necrosis factor α; t-PA, tissue-type plasminogen activator; VEGF, vascular endothelial growth factor.

**Table 2:** ECM components involved in angiogenesis.

Proangiogenic Factor		Antiangiogenic Factor	
Factor	Biological Effect	Factor	Biological Effect
Collagen IV	Low dose: neovessel elongation and survival ↑, high dose: neovessel stability ↑	Endostatin	Proliferation and migration of endothelial cells ↓, adipogenesis ↓
Laminin	Proliferation and migration of endothelial cells ↑	Arresten, canstatin, and tumstatin	Proliferation and migration of endothelial cells ↓, microvessel density ↓, VEGF ↓
Fibronectin	Migration and survival of endothelial cells ↑, number of microvascular cells ↑		
Hyaluronan fragments	Proliferation and migration of endothelial cells ↑, bFGF-induced neovascularization ↑	Laminin fragments	Tube formation and migration of endothelial cells ↓, apoptosis of endothelial cells ↑
Osteopontin	VEGF release ↑, migration and tube formation of endothelial cells ↑	Fibronectin and anastellin	Blood vessel density ↓
Elastin and elastokine	Tube formation of endothelial cells ↑, ProMMP-2 expression and activity ↑	Hyaluronan	Proliferation, migration, and capillary tube formation of endothelial cells ↑

**Table 3:** Effects of angiogenesis modulators on angiogenesis and obesity.

Angiogenesis Modulator	Angiogenesis	Obesity	Mouse Model
TNP-470	Methionine aminopeptidases ↓, proliferation of endothelial cells ↓, angiogenesis ↓, vascular growth ↓	Body weight ↓, proliferation of 3T3-L1 preadipocytes ↓	HFD-fed, A^y^, Cpe^fa^t, and *ob/ob* mice
CKD-732 (TNP-470 analogue)	Methionine aminopeptidases ↓, proliferation of endothelial cells ↓	Body weight ↓, fat mass ↓, adipocyte size ↓	Arcuate nucleus lesion and *ob/ob* mice, SD rats, and OLETF rats
Galardin	MMP activity ↓	Gonadal adipose tissue mass ↓	HFD mice
BAY 12-9566	MMP activity ↓, bFGF-induced angiogenesis ↓	Body weight ↓	*Ob/ob* mice
Thalidomide	bFGF-induced angiogenesis ↓, neovascularization ↓	Body weight ↓	*Ob/ob* mice
VEGFR blockers	Angiogenesis ↓, fat vessel ↓	Adipogenesis ↓, fat tissue expansion ↓, body weight ↓	HFD mice

bFGF, basic FGF; FGF, fibroblast growth factor; HFD, high-fat diet; MMP, matrix metalloproteinase; OLETF, Otsuka Long-Evans Tokushima fatty; SD, Sprague Dawley; VEGF, vascular endothelial growth factor; VEGFR, VEGF receptor.

**Table 4:** Effects of medicinal herbs on angiogenesis and obesity.

Angiogenesis Modulator	Angiogenesis	Obesity	Mouse Model
Curcumin (polyphenol)	Microvessel density ↓, VEGF and VEGFR expression ↓	Adipogenesis ↓, body weight ↓	HFD mice
EGCG (catechin in green tea)	Tube formation of endothelial cells ↓, VEGF signaling ↓	Body weight ↓, fat mass ↓	HFD mice
Ginseng and ginsenosides	MMP activity ↓, fat vessel ↓, expression of MMP, VEGF-A, FGF-2 ↓	Adipogenesis ↓, body weight ↓	HFD and *db/db* mice
Ob-X (herbal composition from lemon balm, white mulberry, and injin)	Tube formation ↓, VEGF-induced microvessel outgrowth ↓, fat vessel ↓, MMP activity ↓	Adipogenesis ↓, body weight ↓, adipose tissue growth ↓	HFD and *ob/ob* mice
ALS-L1023 (lemon balm)	Tube formation ↓, VEGF- and FGF-induced endothelial cell proliferation ↓, fat vessel ↓, MMP activity ↓	Adipogenesis ↓, adipose tissue mass ↓, body weight ↓	HFD mice

EGCG, epigallocatechin gallate; FGF, fibroblast growth factor; HFD, high-fat diet; MMP, matrix metalloproteinase; VEGF, vascular endothelial growth factor.
